# Design and Development of a Hair-like Sensor with Bridge-Type Flexible Amplification Mechanisms

**DOI:** 10.3390/s23177354

**Published:** 2023-08-23

**Authors:** Yongzhen Li, Pei Cao, Peng Zhang, Hua Yang, Xiaofeng Zhu, Ruihua Guo

**Affiliations:** 1 Institute for Smart Ageing, Beijing Academy of Science and Technology, Beijing 100089, China; yongzhen_l@sina.com (Y.L.); caopei2022@163.com (P.C.); yanghua@bjast.ac.cn (H.Y.); zxf_402@163.com (X.Z.); 2 National Center for Occupational Safety and Health, National Health Commission of the People’s Republic of China, Beijing 102308, China; zhangpengbd@163.com

**Keywords:** hair-like sensor, bridge-type amplification mechanism, resonator, airflow rate

## Abstract

Compared with lever-type amplification mechanisms, bridge-type flexible amplification mechanisms have advantages in terms of amplification ratio and structural compactness. Therefore, they can effectively replace the lever-type amplification mechanism in the existing hair-like sensors and realize the development of miniature hair-like sensors with high sensitivity. With that in mind, a highly sensitive hair-like sensor based on a bridge-type amplification mechanism with distributed flexibility is presented to measure the airflow rate. First, the structural composition and operating principle of the hair-like sensor are described. Then, detailed design and analysis of the hair-like sensor are carried out, focusing on the design of the hair post structure, amplification mechanism, and resonator. Furthermore, the designed hair-like sensor is processed and prepared, and some experimental studies are conducted. The experimental results demonstrate that the developed hair-like sensor can measure the airflow rate with high sensitivity up to 8.56 Hz/(m/s)^2^. This provides a new concept for the structural design of hair-like sensors and expands the application of bridge-type flexible amplification mechanisms in the field of micro/nano sensors.

## 1. Introduction

**Figure 1:**
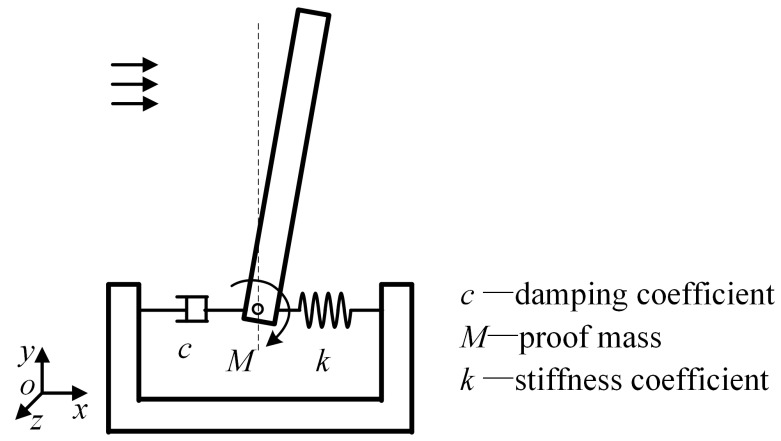
Schematic diagram of the principle.

Inspired by the idea of bionics, there has been wide interest in research into hair-like sensors because of their advantages such as high sensitivity and wide measuring range [1,2,3,4]; their working principle is shown in Figure 1. The hair-like sensor can realize the effective measurement of linear/angular velocity, acceleration, and other information so that it can be widely used in lateral line systems, inertial navigation systems, flow sensing, situation awareness, and so forth. According to the different realization principles, the hair-like sensor can be divided into piezo-resistive [5,6,7,8], capacitive [9,10,11], piezoelectric [12,13], resonant [14,15,16,17,18,19], and so on [1,3,4]. Resonant sensors can realize quasi-digital signal output, which can effectively avoid the problem of low measurement accuracy caused by analog-to-digital conversion.

Therefore, resonant hair-like sensors are favored by many researchers. Yang et al. [14,15,16,17,18,19] developed several hair-like sensors based on the resonance principle, which can realize the measurement of multiple physical uniaxial and biaxial quantities. However, micro lever-type amplification mechanisms are widely used in the existing hair-like sensors, which realize force amplification based on lumped compliance. They have the problem of stress concentration, especially for micro/nano-mechanical structures made from silicon-based materials, with which it is easy to cause structural damage [20,21,22]. Moreover, a large actuation force is required, so it is difficult to develop a highly sensitive hair-like sensor. The bridge-type amplifier composed of flexible hinges with distributed compliance can also be used to amplify the effective force or displacement. The magnification ratio of the lever-type amplifier is related to the lever arm size, so it is not easy to realize miniaturization for the application of a high magnification ratio, while the bridge-type amplifier has the advantages of compact structure and large amplification ratio which is related to its tilt angle. There are many research and application achievements of bridge-type amplifiers, and gratifying research results have been obtained [23,24,25].

In our previous work [26,27], an accurate theoretical model of bridge-type amplifiers is established and its application is studied. During this process, the advantages of a large amplification ratio and compact structure of bridge-type amplifiers are fully explored, which can be applied to the research of hair-like sensors. Therefore, a hair-like sensor based on the bridge-type amplifier will be developed. On the one hand, it can explore the design requirements of bridge-type amplifiers in MEMS structures. On the other hand, it can enrich the design types of hair-like sensors and expand the application of bridge-type amplifiers in the MEMS field.

In this paper, a resonant hair-like sensor based on a two-stage bridge-type amplifier is developed. Firstly, the structure composition and working principle of the hair-like sensor is introduced in detail. Then, a detailed design of the sensor is carried out, particularly on the hair post structure, mechanical structure, and resonator. Furthermore, a specific analysis is conducted on the preparation process of the sensor, and the processing and assembly of the sensor are completed. Finally, an experimental study is conducted on the measurement of airflow rate with the hair-like sensor.

## 2. Mechanism Description

**Figure 2:**
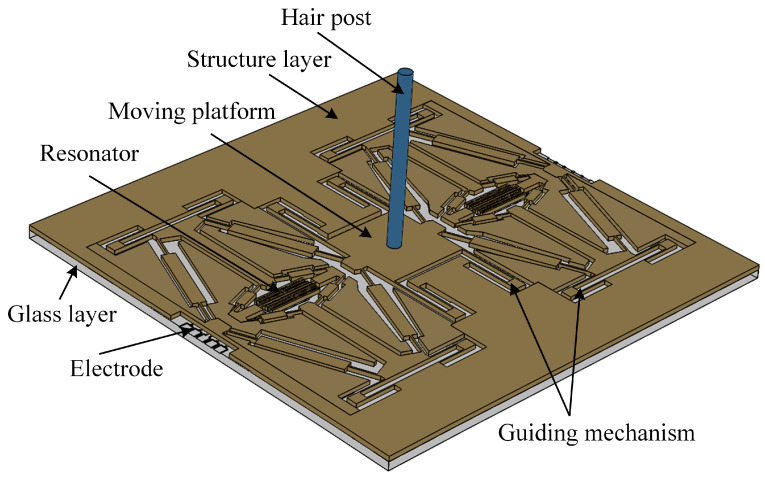
Structure of the hair-like sensor.

According to the working principle of the hair-like sensor, its root generates a certain torque when external interference acts on the hair post structure. To improve the detection accuracy of the sensor, the torque is amplified by an amplifying mechanism and then applied to the shaft end of the resonator. Then, the magnitude of the external interference signal is obtained by detecting the change in the resonant frequency of the resonator. Therefore, the hair-like sensor mainly consists of hair post structure, amplification mechanism, resonator, and other components. The resonator adopts the double-ended tuning fork (DETF) type and its resonant frequency is affected by the change in axial force. To further improve the measurement accuracy of the hair-like sensor, a differential detection method is adopted. When one resonator is subjected to axial tension, the other resonator is subjected to axial pressure by using two resonators. In theory, the variation amplitude in the resonant frequency of the two resonators is equal. The amplification mechanism plays an important role in improving the detection accuracy of the sensor. That is, the amplification mechanism should have a large force amplification ratio. The structural composition of the hair-like sensor is shown in Figure 2.

**Figure 3:**
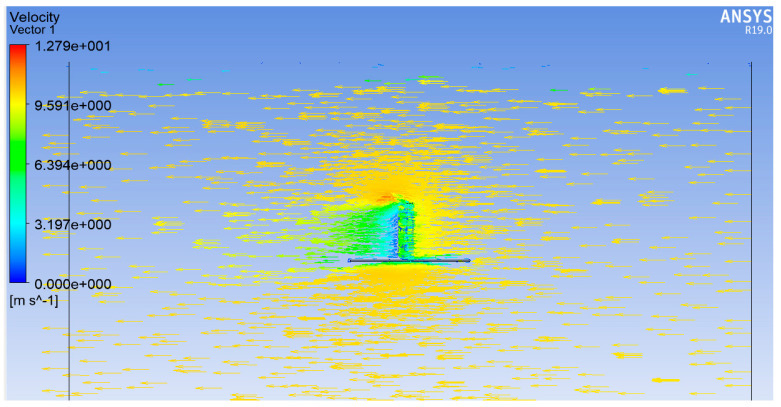
The flow field characteristics of the hair-like sensor.

The simulation analysis results of the hair post in the airflow field are shown in Figure 3. According to conventional fluid mechanics, the hair post is subjected to the drag force generated by the airflow field. The drag force is mainly attributed to the pressure difference caused by the velocity difference between the front and the back area of the hair post. It can be expressed as

(1) $$F=\frac{1}{2}C\rho v^{2}A=\frac{1}{2}C\rho v^{2}dh$$

where *C* is the drag coefficient, *ρ* is the air density, *v* is the velocity of air, *A* is the area of hair post in the direction of airflow, and *d* and *h* are the diameter and height of the hair post, respectively. 

Then, the force acting on the resonant beams of the DETF can be obtained

(2) $$F_{R}=\xi RF$$

where *ξ* represents the attenuation coefficient and *R* denotes the amplification ratio of the amplification mechanism.

When the resonant beam is subjected to axial force *F_R_*, the natural frequency of the DETF is

(3) $$\omega =\sqrt{\frac{K_{e}+\delta F_{R}}{M_{e}}}$$

where *δ* is the constant associated with structural parameters, and *K_e_* and *M_e_* are the equivalent stiffness and mass of the tuning fork, respectively. 

Before and after the resonant beam is acted on by axial force, the resonant frequency change of the resonator can be expressed as

(4) $$\Delta \omega =\sqrt{\frac{K_{e}}{M_{e}}}-\sqrt{\frac{K_{e}+\delta F_{R}}{M_{e}}}$$

By Taylor series expansion, the resonant frequency variation of the resonator can be approximated as

(5) $$\Delta \omega =\pm \frac{1}{2}\omega F_{R}t$$

According to Equation (5), the resonant frequency changes in direct proportion to the square of the airflow rate. By measuring the variation of the resonant frequency of the resonator, the magnitude of the airflow rate can be obtained. The performance of the sensor is determined by the hair post structure, amplification mechanism, and resonator. In particular, it is affected by the amplification ratio of the amplification mechanism.

## 3. Design and Analysis of Hair-like Sensor

### 3.1. Design of Hair Post Structure

The available types of hair post structures include cylindrical, hollow cylindrical, rectangular, etc. In this paper, the common circular hair post is used, as it is easy to obtain. And the effect of the hair post structure type on the performance of the hair-like sensor is not considered. Refs. [15,17] indicate that the frequency output sensitivity of the sensor increases with the increase in the diameter and height of the hair post. The sensitivity of the hair-like sensor can be improved by optimizing the dimensions of the hair post. The hair post structure is fixed together with the moving platform in the structural layer.

### 3.2. Design of the Amplification Mechanism

**Figure 4:**
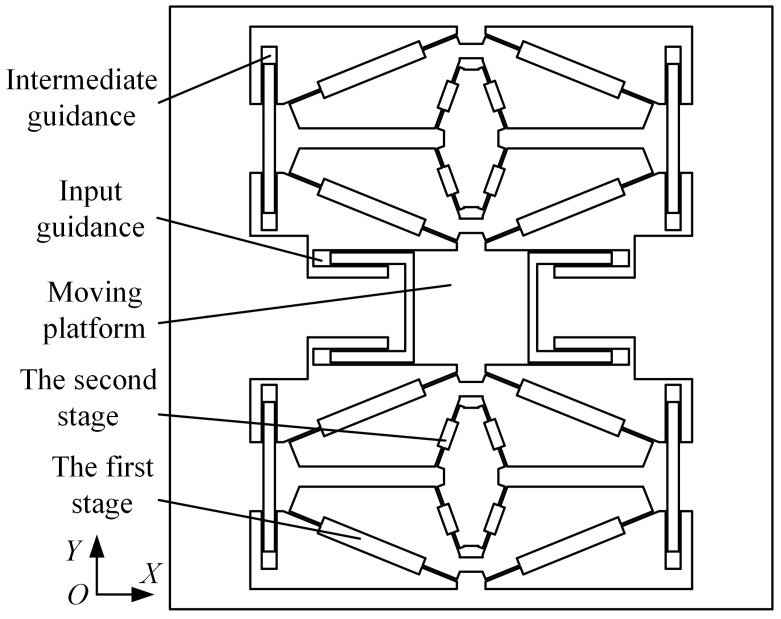
Two-stage amplification and guiding mechanism.

The amplification mechanism is mainly used to amplify the effective force generated by the external signal. And then the amplified effective force is applied to the DETF resonator. Common flexible amplification mechanisms include lever-type, bridge-type, and composite-type. The amplification ratio of a lever-type amplification mechanism is related to its arm’s length and the implementation of a large amplification ratio usually results in a large structural size. Because of this, a bridge-type amplification mechanism with a large amplification ratio is selected as the amplification mechanism in the hair-like structure. In order to obtain a larger amplification ratio, two bridge-type amplification mechanisms are used in series to construct a two-stage amplification mechanism. Due to the use of differential detection and the ease of achieving structural symmetry, two sets of two-stage amplification mechanisms are used, as shown in Figure 4.

In a hair-like sensor, the silicon wafer is used as processing material. It is also proved that the elastic modulus of the silicon beam specimen does not show a size effect. And the linear elastic theory is still applicable to the micro flexible mechanism presented based on silicon material in some works of literature [28,29]. The amplification ratio of the amplification mechanism is defined as the ratio of the output displacement to the input displacement. Therefore, the amplification ratio of the two-stage bridge-type amplification mechanism can be expressed as [26]

(6) $$R_{T}=\frac{-L_{1}\left[k_{11}^{\left(1\right)}L_{1}^{3}f_{\mathrm{in}}\cos\alpha_{1}\sin\alpha_{1}\left(1+\cos^{2}\alpha_{2}\right)-4k_{11}^{\left(0\right)}\cos\alpha_{2}\left(L_{1}^{3}\cos^{2}\alpha_{1}+L_{2}^{3}\sin^{2}\alpha_{2}\right)\right]}{\tan\alpha_{1}\sin\alpha_{2}\left[4k_{11}^{\left(0\right)}\left(L_{1}^{3}\cos^{2}\alpha_{1}+L_{2}^{3}\sin^{2}\alpha_{2}\right)-k_{11}^{\left(1\right)}L_{1}L_{2}^{2}f_{\mathrm{in}}\cos\alpha_{1}\sin\alpha_{1}\cos\alpha_{2}\right]}$$

where *L*_1_ and *L*_2_ are the lengths of the beam flexure in the first and second stage amplifier, respectively; *α*_1_ and *α*_2_ are the inclined angles of the first and second stage amplifier; and the coefficients *k* are the stiffness coefficients, which have been given out in Ref. [30]. All non-dimensionalized quantities are represented by lower-case letters, while their corresponding dimensional parameters are denoted in upper case. The relationship between force amplification and displacement amplification is reciprocal. In this paper, the force amplification function of the amplifying mechanism is used. That is, the output displacement is less than the input displacement.

**Figure 5:**
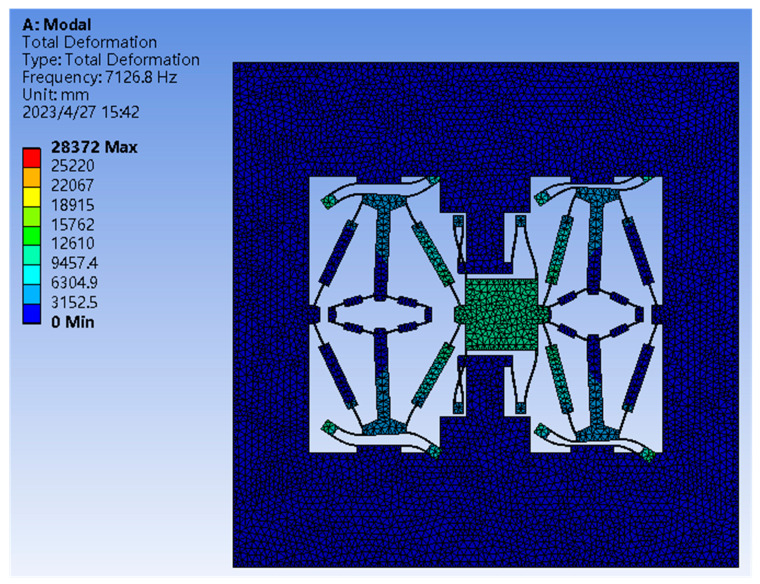
The first mode of two-stage amplification and guiding mechanism.

Due to the use of the amplification mechanism with distributed flexibility, guiding structures need to be designed at the input and output ends of the first-stage amplification mechanism to obtain the desired first-mode shape, while the output end of the second-stage amplification mechanism is connected to the DETF resonator, thereby applying the amplified effective force to the shaft end of the resonant beam. The guiding structure adopts a parallel parallelogram flexure, which achieves an excellent guiding effect by adjusting the length and width of the flexure. The input guidance connects the moving platform and anchor point to ensure that the moving platform moves in the *Y* direction. The intermediate guidance is mainly used to prevent complex motion of the amplification mechanism due to the large distribution flexibility. The modal analysis of the main structure is conducted using the finite element analysis method and the results are shown in Figure 5. It can be seen that the first mode of the main structure is the desired motion.

### 3.3. Design of Double-Ended Tuning Fork Resonator

**Figure 6:**
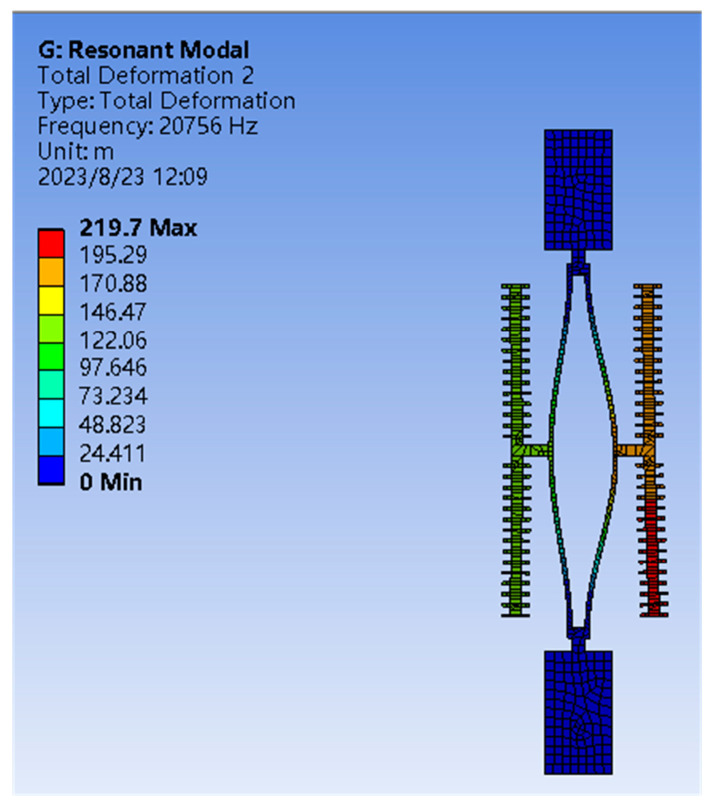
The anti-phase resonant frequencies of resonators.

With the advantages of high-quality factor, strong anti-interference ability, and simple structure, the double-ended fixed tuning fork (DETF) resonator is widely used in MEMS devices. In this paper, two DETF resonators are used as the core detection unit for external physical information. To more accurately detect external physical information, a differential method is used to detect changes in the frequency amplitude of the resonator. The primary task is to determine the resonant frequency of the resonator. The modal analysis of DETF is conducted by using the finite element analysis method and the results are shown in Figure 6. It can be seen that the anti-phase resonant frequency of the resonator is 20.7 KHz, which is affected by the axial force acting on the resonant beam. The axial force is the effective force after external interference information passes through the hair post structure and bridge-type amplification mechanism.

## 4. Fabrication of the Hair-like Sensor

According to the structural design and analysis results of the hair-like sensor, the key structural parameters of the designed sensor are shown in Table 1.

**Figure 7:**
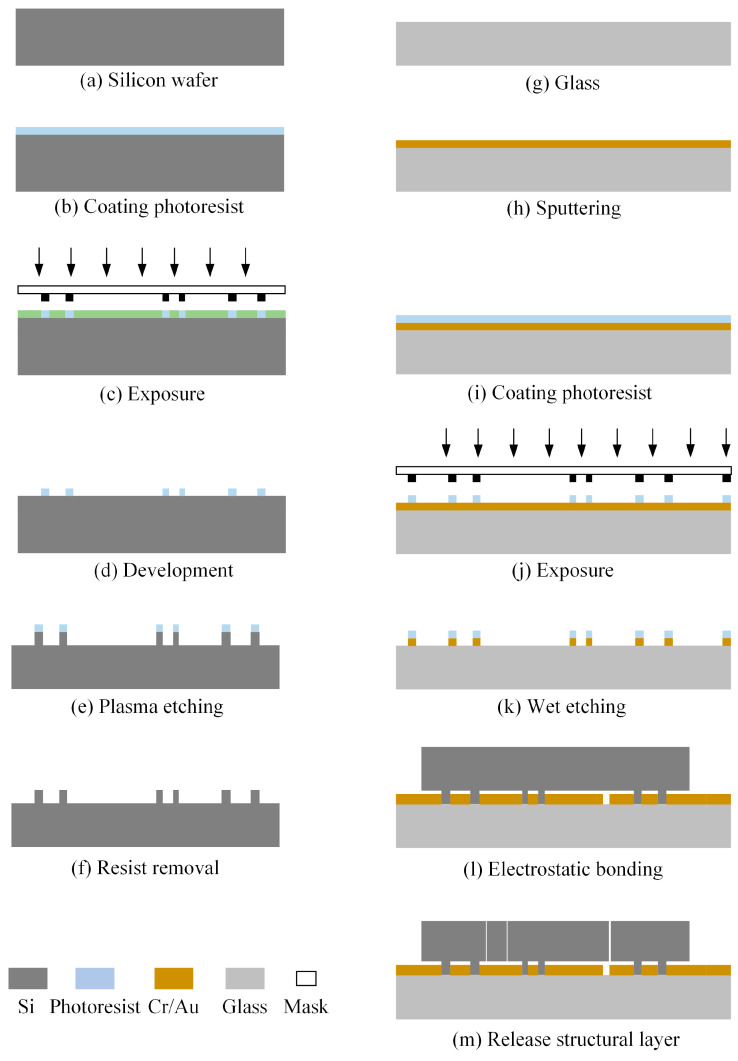
The fabrication process of the sensor.

Based on the structure of the designed hair-like sensor, the preparation process of the sensor is analyzed. Considering the difficulty of integrating hair post structure and main structure for processing, a composite process of micro-assembly is used to prepare the sensor. The main mechanical structure of the sensor is realized by deep reactive ion etching technology. The mono-crystalline silicon material is used for the preparation of the mechanical structure layer, including moving platforms, guiding structures, bridge-type amplification structures, and resonators. Because of the small comb gap of the DEFT fork resonator, the etching depth of the silicon wafer is limited to a certain extent, which is used to guide the design of the sensor structure. The bridge-type amplification structure and guiding structure have many thin-walled structures in the structural layer, which require extremely high requirements for deep reactive ion etching technology. For this reason, a smaller etching area is designed near the thin-walled structure, increasing the portion that needs to be retained. A borosilicate glass is selected as the substrate and signal lines are prepared on its surface. Then, the two layers of structure are bonded together through anodic bonding. Finally, the silicon wafer is thinned and profiled, and the sensor structure is released using a deep reaction ion etching process. The step-by-step fabrication process of the structure and signal layer is shown in Figure 7. The sensor is packaged using a ceramic shell, and the electrodes and pins are connected through gold wires. The existing ABS material solid round rod or hollow tube is selected as the hair post structure, and the required length can be cut. The micro-assembly of the system is completed by bonding the hair post structure and the main structure. Then, the sensor is finally packaged using a metal cover plate. The packaged hair-like sensor can be soldered to the PCB board for driving testing.

**Figure 8:**
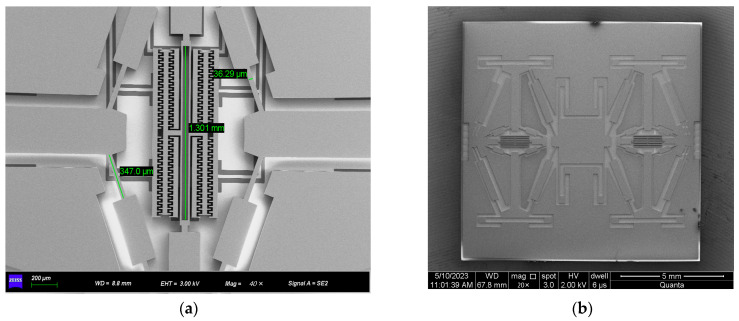
An SEM image of the mechanical structure layer: (**a**) local enlarged drawing; (**b**) whole structure diagram.

The hair-like sensor is characterized by a scanning electron microscope (SEM), as shown in Figure 8. The maximum error of the main structural dimensions is not greater than 10 μm, which is approximately a 3.3% deviation from the designed value.

## 5. Experimental Evaluation

**Figure 9:**
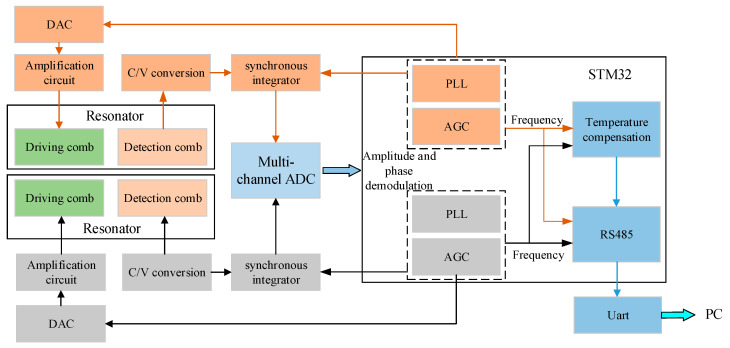
Scheme of the resonator drive and capacitance detection.

**Figure 10:**
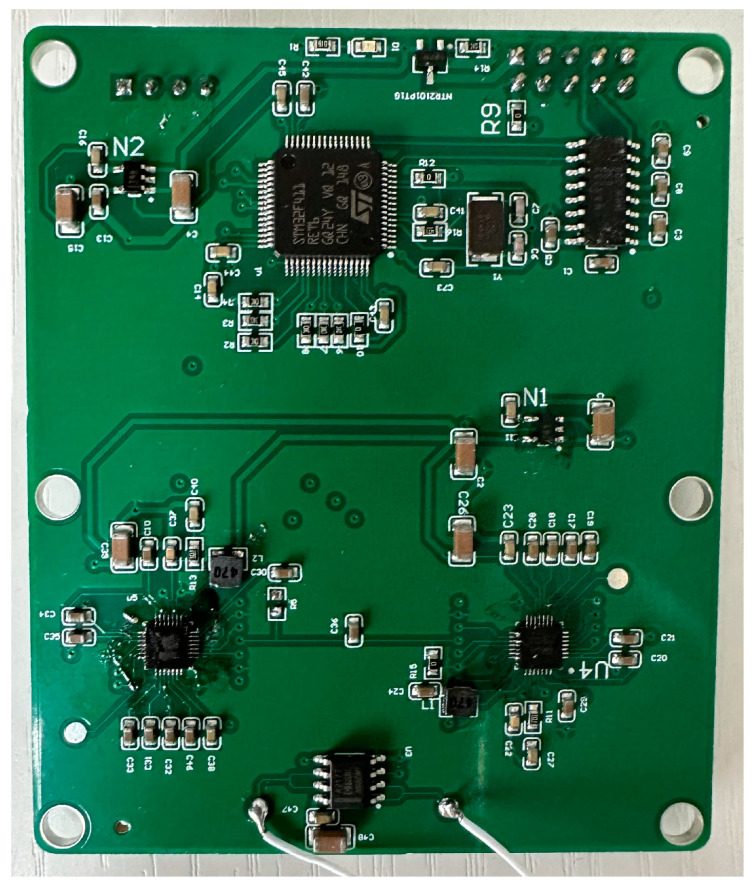
Sensor drive and detection system.

According to the working principle of the hair-like sensor, it is necessary to design a resonator drive and capacitance detection system, as shown in Figure 9. This system can provide a sinusoidal driving signal that quickly locks in the resonant frequency variation of the resonator. In addition, the capacitance–voltage conversion circuit is used to collect data on the capacitance changes caused by the motion of the resonant beam. Based on the specific performance of the prepared hair-like sensor, a sensor-driven oscillation circuit board is designed (as in Figure 10). The packaged hair-like sensor is soldered onto the PCB board.

**Figure 11:**
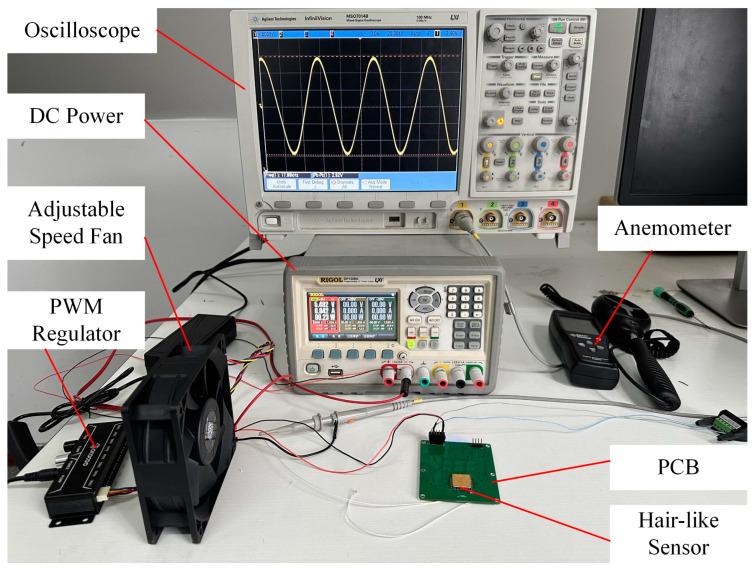
Experimental test platform.

To test the performance of the hair-like sensor, an airflow rate test is carried out. A DC brushless fan is adopted to provide wind speeds of different sizes, which can be adjusted by PWM. And the wind speed of the fan can be calibrated using an anemometer. The resonant frequency and vibration waveform of the resonator are collected by oscilloscope. Then, the relationship between the frequency and the wind speed can be established. The objects of the experiment setup used to characterize the performances of the hair-like sensor are shown in Figure 11. 

**Figure 12:**
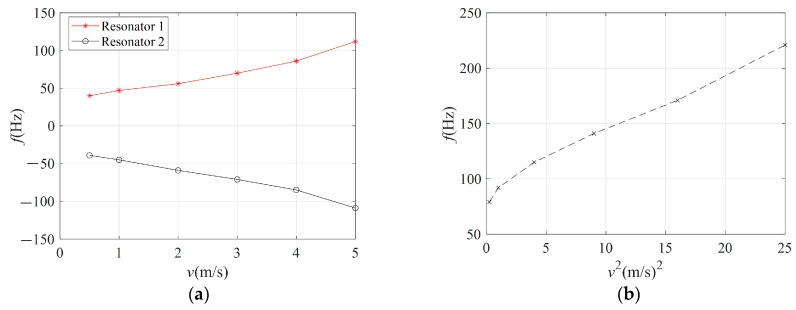
Experimental results: (**a**) frequency change of two resonators at different airflow rates; (**b**) total variation of two resonators at different airflow rates.

Under different wind speed conditions, the hair-like sensor is tested and studied. Constant wind speed is generated by an adjustable fan and calibrated by an anemometer. The response results of two sets of resonators in the sensor are illustrated in Figure 12. When the hair post structure is disturbed by the airflow, one of the resonators is subjected to pressure and the other is subjected to tension. And then the resonance frequency of the two resonators changes, as shown in Figure 12a. When the resonant beam in the resonator is subjected to tension or pressure, the amplitude of its resonant frequency increases. The higher the airflow rate, the greater the effective force on the resonant beam. Therefore, as the airflow rate increases, the amplitude of the resonant frequency also increases. The total amplitude of the resonant frequency change of the two resonators at different airflow rates is shown in Figure 12b. It can be calculated from the experimental results that the sensitivity of the hair-like sensor is approximately equal to 8.56 Hz/(m/s)^2^. Of course, it is not completely constant and does not exactly agree with the theoretical results. In theory, the change in resonant frequency is proportional to the square of the airflow rate (refer to Equation (5)). On the one hand, it is due to the numerical deviation between the theoretical and experimental results. And on the other hand, it is caused by the actual nonlinearity (material nonlinearity, data acquisition circuit nonlinearity, etc.). The possible reasons are speculated as follows:

(a) The machining of the mechanical layer structure causes certain errors, especially the comb, bridge-type amplification structure, and guiding mechanism. The limitation of the existing etching technology on the depth of the structure affects the design of the thickness of the structural layer. The thickness of the structure layer has a great influence on the working mode of the system.
(b) The size deviation of hair post structure and the accuracy of installation position all have a certain influence on the experimental results.
(c) The experimental results are also influenced by the circuit design of the resonator drive and resonant frequency signal acquisition.

In addition, the minimum airflow rate of this experiment is set at 0.5 m/s and no smaller airflow rate test is conducted. On the one hand, it is limited by the small airflow rate generator and, on the other hand, it is necessary to have a vacuum test environment to reduce the influence of ambient airflow on the experimental results. Compared with the sensor in Reference [15], the sensitivity of the developed sensor is 15.5% lower than that of the sensor. And the length of the hair post structure used in this paper is only 5 mm, which is much smaller than that of the hair post structure. It can be seen that the performance of the developed hair-like sensor has great competitiveness in similar products. Of course, its performance can be further improved.

## 6. Conclusions

According to the working principle of the hair-like sensor, the structure composition of a new hair-like sensor with bridge-type amplification mechanisms is determined. The key components that affect the performance of the sensor are the hair post structure, the amplification mechanism, and the resonator, especially the amplification mechanism. The three units are designed and analyzed in detail, and the overall design of the hair-like sensor is completed. For high consistency, a common ready-made cylindrical hair post structure is used. The bridge-type amplifier with the advantages of compact structure and large magnification ratio is used to amplify the effective force. A parallel parallelogram flexure structure is designed as a guiding mechanism. The modal analysis of the mechanical structure layer is carried out by the finite element method and the first mode of the structure is the working mode, which also proves the rationality of the designed structure. 

Based on the theoretical guidance and numerical analysis, a hair-like sensor is prepared and tested. It can be seen from experimental results that the developed hair-like sensor can measure the airflow rate with high sensitivity up to 8.56 Hz/(m/s)^2^. A new design idea of a hair-like sensor based on a bridge-type amplification mechanism is presented. Under the premise of ensuring the high sensitivity of the sensor, it also has a smaller outline size. In the future, we will focus on improving the performance of hair-like sensors and convenient data acquisition.

## Figures and Tables

**Table 1:** Key parameters of the prepared hair-like sensor.

Parameters	Values
Diameter of the hair post (mm)	1
Height of the hair post (mm)	5
Length of the resonant beam (mm)	1320
Width of the resonant beam (mm)	0.02
Thickness of the structure layer (mm)	0.1
Width of the flexure beam (mm)	0.04
Inclined angle of the amplifier (°)	20
Clearance of the comb (mm)	0.01
Number of driving comb	30

## Data Availability

Not applicable.

## Abbreviations

The following abbreviation is used in this manuscript:

DETF	Double ended fixed tuning fork
